# Can functional genomic diversity provide novel insights into mechanisms of community assembly? A pilot study from an invaded alpine streambed

**DOI:** 10.1002/ece3.7973

**Published:** 2021-08-04

**Authors:** Hannah E. Marx, Marta Carboni, Rolland Douzet, Christophe Perrier, Franck Delbart, Wilfried Thuiller, Sébastien Lavergne, David C. Tank

**Affiliations:** ^1^ Department of Biology & Museum of Southwestern Biology University of New Mexico Albuquerque New Mexico USA; ^2^ Department of Sciences Università Roma TRE Roma Italy; ^3^ CNRS Lautaret Jardin du Lautaret Université Grenoble Alpes Grenoble France; ^4^ Laboratoire d'Ecologie Alpine (LECA) CNRS Université Grenoble Alpes Université Savoie Mont Blanc Grenoble France; ^5^ Department of Biological Sciences University of Idaho Moscow Idaho USA; ^6^ Institute for Bioinformatics and Evolutionary Studies University of Idaho Moscow Idaho USA; ^7^ Stillinger Herbarium University of Idaho Moscow Idaho USA; ^8^ Present address: Department of Botany and Rocky Mountain Herbarium University of Wyoming Laramie WY 82072‐3165 USA

**Keywords:** alien, alpine, community genomics, functional trait, gene expression, invasive, RNA‐seq, transcriptome

## Abstract

An important focus of community ecology, including invasion biology, is to investigate functional trait diversity patterns to disentangle the effects of environmental and biotic interactions. However, a notable limitation is that studies usually rely on a small and easy‐to‐measure set of functional traits, which might not immediately reflect ongoing ecological responses to changing abiotic or biotic conditions, including those that occur at a molecular or physiological level. We explored the potential of using the diversity of expressed genes—functional genomic diversity (FGD)—to understand ecological dynamics of a recent and ongoing alpine invasion. We quantified FGD based on transcriptomic data measured for 26 plant species occurring along adjacent invaded and pristine streambeds. We used an RNA‐seq approach to summarize the overall number of expressed transcripts and their annotations to functional categories, and contrasted this with functional trait diversity (FTD) measured from a suite of characters that have been traditionally considered in plant ecology. We found greater FGD and FTD in the invaded community, independent of differences in species richness. However, the magnitude of functional dispersion was greater from the perspective of FGD than from FTD. Comparing FGD between congeneric alien–native species pairs, we did not find many significant differences in the proportion of genes whose annotations matched functional categories. Still, native species with a greater relative abundance in the invaded community compared with the pristine tended to express a greater fraction of genes at significant levels in the invaded community, suggesting that changes in FGD may relate to shifts in community composition. Comparisons of diversity patterns from the community to the species level offer complementary insights into processes and mechanisms driving invasion dynamics. FGD has the potential to illuminate cryptic changes in ecological diversity, and we foresee promising avenues for future extensions across taxonomic levels and macro‐ecosystems.

## Graphical abstract

Functional trait diversity patterns have been central for understanding how communities respond to change. We explore the diversity of functional genomic traits in an invaded alpine ecosystem, exposing cryptic functional diversity that helps disentangle controversial hypotheses of invasion biology and community assembly.

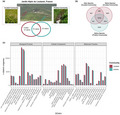

## 1 INTRODUCTION

Species invasions have become an important study system for understanding community assembly (Pearson et al., 2018; Strauss et al., 2006) and eco‐evolutionary feedbacks (Colautti et al., 2017; Strauss, 2014), due to the ubiquity, replication, and detailed records of invasions and invaded systems at different scales around the globe (Sax et al., 2007). Species invasions can also be used to test evolutionary hypotheses of trait evolution, because the environments selecting on traits, as well as sister lineages, are still present today (Lu‐Irving et al., 2018). Both experimental (Pearson et al., 2012) and observational studies (Pyšek & Richardson, 2006; Rejmánek, 1996; Rejmanek & Richardson, 1996) have shown that alien species often differ from natives in phenotypic functional traits (van Kleunen et al., 2010; Pyšek & Richardson, 2008) and that specific functional traits are associated with invasive plants (Leishman et al., 2007; Pearson et al., 2012; Pyšek & Richardson, 2006; Rejmanek & Richardson, 1996). In particular, while similarity in traits with native species may in some cases promote the naturalization of alien species, distinct traits appear to promote invasiveness (Divíšek et al., 2018). For instance, specific leaf area, plant stature (height, canopy extent), and leaf nitrogen content are often invoked to separate species along a trade‐off of resource acquisition versus stress resistance (e.g., Lavergne et al., 2003, 2004). Quantifiable measures of such functional trait diversity may be compared across ecosystems (Petchey & Gaston, 2002) and have been increasingly used to identify generalizable processes promoting ecological diversity and ecosystem functioning (Cadotte et al., 2011; Carmona et al., 2016; McGill et al., 2006; Wilcox et al., 2018), as well as community assembly (Chalmandrier et al., 2015, 2017; Mouchet et al., 2010; Pavoine & Bonsall, 2011).

Because it is impossible to measure all phenotypic traits across even a modest‐sized community, studies of functional trait diversity have relied on a few measurable traits as a sufficient proxy for underlying and unmeasured functional diversity (Lavorel & Garnier, 2002; Westoby & Wright, 2006). For example, studies of trait‐based plant ecology have primarily focused on quantifying vegetative traits, and reproductive or dispersal traits have much less frequently been measured and used to study functional diversity patterns (Lu‐Irving et al., 2018). Yet, we know functional trait space is multidimensional and challenging to quantify (Laughlin, 2014; Maire et al., 2015; Violle et al., 2007), and it is not always clear which traits are ecologically relevant because functional relevance can be contingent on the environmental setting (Cooper et al., 2010; Forrestel et al., 2017; Gould et al., 1979). Additionally, niche differences that stabilize coexistence may result from specific combinations of traits (Kraft et al., 2015), so decisions to deduce a certain trait set could mislead interpretations of diversity patterns.

Here, we present the results of a pilot study intending to extend the examination of community diversity patterns across additional trait axes by quantifying community transcriptomic diversity (functional genomic diversity, FGD). Transcriptomes describe the total diversity of expressed genes (transcripts) in any biological organ or individual, and can now be studied in nonmodel organisms in a given macro‐ecosystem context (da Fonseca et al., 2016; Strickler et al., 2012). We posit that measuring FGD from transcriptomes has the potential to overcome many biases and assumptions of traditional measures of functional trait diversity described above. To connect back with meaningful biological functions, transcripts can be assembled into orthologous genes (related by descent from a common ancestor) and annotated by comparison with curated genomes. These genes can then be assigned to protein functional categories (e.g., housekeeping, metabolic functions) using online repositories of gene ontologies and gene networks (Segata & Huttenhower, 2011). Annotations of transcribed genes that are unique to certain species, clades, or habitats may identify unique functional diversity (unknown traits) important for a particular ecosystem at a specific point in time. For microbial taxa, transcribed gene products have long been used as “functional traits” to characterize communities (Damon et al., 2012; Urich et al., 2008). Such studies have paved the way toward a genetic perspective on diversity dynamics using environmental sequencing to catalog all the genes, or transcribed gene products, across ecosystems ranging from the human gut to arctic soils, illuminating the important products of ecosystem function, and defining healthy communities (Raes & Bork, 2008). While functional genomic approaches are frequently applied to microbial ecosystems (Morgan et al., 2013; Poole et al., 2012), application of these methods to macro‐ecosystems remains largely unexplored (Lee et al., 2011; Zambrano et al., 2017). However, functional genes describe nearly all products of an organism in its environment and therefore may provide more accurate insights into the relevant phenotypes important for the ecological role or dynamics of a species—such as its invasiveness (Neal Stewart et al., 2009). Also, quantifiable measures of functional genomic traits have the potential to be compared across ecosystems—from plants to microbes—expanding beyond a few measurable functional traits to all underlying expressed diversity in the community. Here, we evaluate whether the extension of functional trait ecology into the measurements of functional genes allows progress toward a better understanding of invasion processes in a plant community.

The few studies beginning to explore transcriptomes of invasive plants have focused on the qualities of specific successful invaders (Broz et al., 2008, 2009), comparisons of several invasive species within a particular family (Lai et al., 2008), introduced and native populations of a single invasive species across its range (Dlugosch et al., 2013; Guggisberg et al., 2013; Hodgins et al., 2013; Lai et al., 2008), or comparisons of expression profiles between alien species and native congeners (Guo et al., 2018). Notable findings include differential expression of stimulus and stress response genes between aliens and natives (Guo et al., 2018), introduced populations with reduced expression of transcripts related to constitutive defense relative to native counterparts (Broz et al., 2009), and differential expression of transcripts specific to competitor species (Broz et al., 2008). However, most of this work has been restricted to a few plant lineages (mostly within the Asteraceae family) and never applied to a whole community context. Yet, by measuring transcriptomic diversity of both alien and native species occurring across an invaded landscape, it is also possible to test controversial hypotheses on the mechanisms through which alien species invade communities and affect native species (Table 1).

**TABLE 1:** Expectations of observed functional diversity patterns at the community or species level depending on the hypotheses of specific mechanisms driving community assembly and alien success assessed by measuring different components of functional diversity

Level of comparison	Hypothesis	Mechanism	FTD measure	FGD measure	Expected FTD pattern	Expected FGD pattern
(a) Invaded versus pristine community	Niche partitioning	Novel functions of invaders fill available niche space	Species‐level measurements of phenotypic traits, including maximum height (m), average leaf number, total leaf area (mm^2^), specific leaf area (mm^2^/mg), leaf dry matter content (mg/g), and leaf nitrogen content (% dry‐leaf mass)	Counts of unigenes overall and functional dispersion (FDis) of unigene counts within each functional annotation category	Higher FTD within the invaded compared with the pristine community	Higher FGD within the invaded compared with the pristine community
	Competitive exclusion	Fitness dominance of invaders leads to native exclusion			Lower FTD within the invaded compared with the pristine community	Lower FGD within the invaded compared with the pristine community
	Matched species replacement	Functional equivalence			Similar FTD within the invaded compared with the pristine community	Similar FGD within the invaded compared with the pristine community
(b) Congeneric alien–native species pairs	Phenotypic divergence	Limiting similarity	Mean trait measurements for each species	Proportion of distinct unigenes identified within each GO slim category (FGD profiles)	Significant differences in mean trait measurements	FGD profiles alien congener ≠ native congener
	Phenotypic convergence	Habitat filtering			No significant differences in mean trait measurements	FGD profiles alien congener = native congener
(c) Native species sampled in both communities	Alien species alter the expression of genes in native species and impact community structure	Trait‐based processes structure relative abundance of native species	Not investigated	Differential expression (DE) of unigenes when species was sampled in the invaded or the pristine community	Not investigated	Positive or negative correlation between DE and difference in relative abundance of native species sampled from the invaded versus the pristine community

Note

At the community level, we measured functional trait diversity (FTD) from a suite of phenotypic characters and functional genomic diversity (FGD) as the count of expressed unigenes that annotated to each of 47 functional Gene Ontology (GO) slim categories. Functional dispersion was then quantified across all species within the invaded and pristine community. Between congeneric alien–native species pairs in the invaded plot, we compared the proportion of unigenes in each GO slim category (FGD profiles). For native species collected from both the invaded and pristine communities, we quantified the change in unigene expression level in response to growing in the presence of alien species. This differential expression (DE) was summarized in two ways: the log 2 fold change in unigene expression level between communities, and the fraction of unigenes that showed statistically significant DE between communities.

To begin to test hypotheses of mechanisms important for invasion success (Table 1a), in this pilot study, we first broadly characterized and described community‐level functional trait diversity (FTD) and functional genomic diversity (FGD) between an invaded and an uninvaded (i.e., pristine) alpine streambed plant community. We quantified FTD and FGD for each species sampled in both plots to have replication at the species level for comparison of trait and transcriptomic analyses. As with community assembly in general, a number of mechanistic hypotheses have been proposed to explain invasions (MacDougall et al., 2009; Tilman, 2004), leading us to expect three alternative patterns. First, the hypothesis of niche partitioning (stabilizing differences) stipulates that species coexistence is due to niche differences (Chesson, 2000) and promotes invasions when colonizing species exhibit novel functions and fill available niche space (Ricklefs, 2010). Under this hypothesis, we expect increased functional diversity in the invaded compared with the pristine community, as alien species should add to, and thereby elevate, trait diversity in the community overall. Alternatively, mechanisms of fitness dominance may drive competitive exclusion, allowing certain introduced species to locally extirpate one or several native species. In this case, we expect comparatively decreased diversity in the invaded community compared with the pristine as alien species decrease functional diversity through local native species extinction. Finally, stable functional diversity may result from matched species replacement, where one alien species replaces one native that is functionally equivalent (Hubbell, 2005; Young et al., 2007), which would result in similar functional profiles in both communities.

Congeneric species pair comparisons (Table 1b) enable direct measurement of the evolutionary stasis or divergence of traits compared with a recent common ancestor (Ackerly, 2003). Therefore, finer functional comparisons between congeneric alien–native pairs offer insights into mechanisms driving alien success (van Kleunen et al., 2010; Ordonez et al., 2010). We may expect phenotypic divergence between alien and native congeners if communities are assembled through a limiting similarity mechanism (Macarthur & Levins, 1967). Under this scenario, successful alien species differ functionally from native species, because this dissimilarity would allow establishment or coexistence, and we should thus expect unique functional profiles in the alien congener. Alternatively, we may expect phenotypic convergence between alien and native congeners if communities are assembled through a mechanism of habitat filtering (e.g., pre‐adaptation to stress tolerance; Diaz et al., 1998; Keddy, 1992; Li et al., 2018) or a mechanism of self‐organized similarity along a niche access that can result from the competition if niche differences are the dominant coexistence mechanism (Scheffer & van Nes, 2006). This leads to the prediction that successful aliens should be functionally similar to the native species. Of course, there is more likely to be a combination of both scenarios, and FGD profiles may highlight nuanced dynamics of functional (dis)similarity across different functional genomic categories.

Experimental studies have shown that the magnitude of gene expression patterns can be altered in response to both abiotic (Shimizu‐Inatsugi et al., 2017) and biotic (Geisler et al., 2013) changes in the environment. Comparisons of differential gene expression between native species occurring in both communities may enable us to assess the degree to which native species respond to the local presence of alien species and how this relates to community composition (Table 1c)—a unique axis of functional diversity that is only recently becoming possible to explore. We hypothesized that if aliens are impacting gene expression of other species in the community, we would observe a change in gene expression (i.e., differential expression, DE) of native species, depending on whether they co‐occur with alien species. We also hypothesized that this change in DE for native species would correlate with the change in their relative abundance between invaded and pristine habitats. A relationship (positive or negative) between DE and change in relative abundance from the invaded to the pristine community would indicate that variation in FGD resulting from the presence of alien species can shape local native community structure.

## 2 MATERIALS AND METHODS

### 2.1 Species selection

**FIGURE 1:**
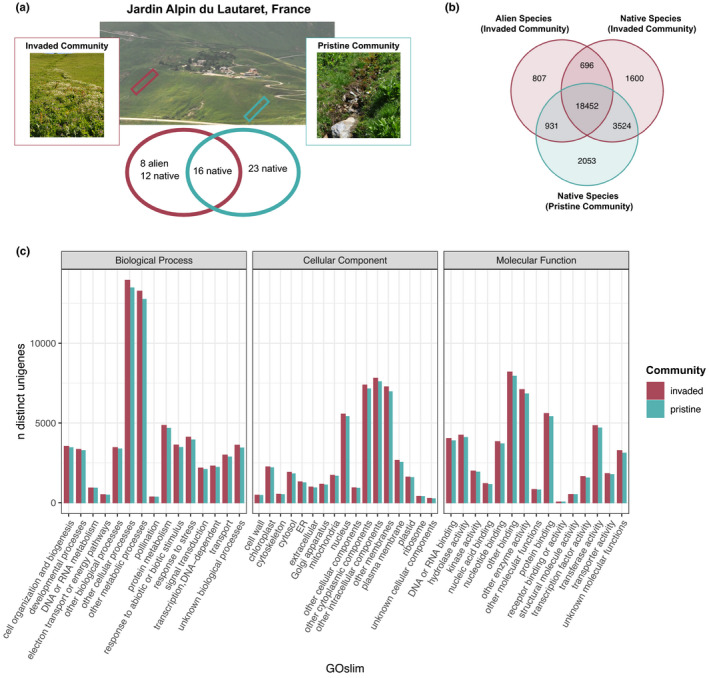
Photographs of the invaded and pristine communities sampled at the Jardin Alpin du Lautaret, France. (a) Venn diagram comparing the observed species richness between the invaded and pristine communities. (b) Venn diagram comparing the functional genomic diversity (FGD) measured by the total number of unigenes from all species sampled from the invaded (*N* = 20; 7 alien and 13 native) and the pristine community (*N* = 16; 10 shared with the invaded community). Species are separated by provenance in the invaded or pristine community to illustrate differences between aliens and natives. (c) The number of distinct unigenes (and gene models) matching each functional GO slim category within the biological process, cellular component, and molecular function aspects recovered from the invaded (pink) or blue (pristine) communities

The Jardin Alpin du Lautaret, France, is an alpine botanic garden located at 2,100 m and manages a living garden of more than 2,000 species from mountain areas around the world. Several plant species introduced into the garden have escaped cultivation and subsequently invaded one stream flowing out of the garden (first observations of escapes in 1920 just after the garden was established), while another stream (about 150 m away) does not flow out from the garden and remains pristine (Figure 1a). Besides invasion, these two riparian sites are perfectly identical in all characteristics including altitude, slope, aspect, soil type, and grazing management, and no fertilizer or other treatment is used in the garden that may promote colonization of the outflowing streambed.

In June 2014, along each invaded and pristine riparian community we performed 20 replicated botanical survey plots of 1 m² organized along an approximately 50‐m linear transect. In each plot, every species was given a score corresponding to its relative spatial cover. Within the pristine and invaded communities, transect abundances were summed across plots for all species. Locally abundant (>10%–25% cover) species were selected for comparisons of functional trait and genomic diversity. Relative abundances for the 28 sampled species were calculated in relation to the other sampled species in each community.

### 2.2 Functional trait diversity (FTD) measurements

For this study, we chose to consider functional traits that have been used previously in explorations of community assembly in invaded systems (Carboni et al., 2016; Marx et al., 2016; Schaefer et al., 2011), and are more generally considered to depict species' ecological strategies along environmental gradients in an alpine context (e.g., Bello et al., 2013). For each species, we recorded maximum height (m) and leaf number in the field from ten distinct individuals sampled along study transects. These same individuals were collected to measure specific leaf area (SLA), leaf dry matter content (LDMC), and leaf nitrogen content (LNC). Total leaf area (LA; mm^2^) was quantified from scans using ImageJ (version 1.51), and fresh mass (g) and dry mass (mg) were recorded from the same leaf. SLA was quantified as the ratio of leaf area (mm^2^) to dry mass (mg), and LDMC (with petioles) was quantified as the ratio of oven dry mass (mg) to water‐saturated wet mass (g). LNC was measured as the mass‐based percentage of nitrogen within leaf tissues (% dry‐leaf mass). To do so, we measured leaf N and C concentrations using classical procedures on an elemental analyzer (Flash EA1112; Thermo Scientific), based on dried and ground leaf material. All measurements were taken following the standardized protocols for functional traits (Pérez‐Harguindeguy et al., 2013). Average values across the ten individuals were used as species‐level phenotypic functional traits.

### 2.3 Functional genomic diversity (FGD) measurements

For the same locally abundant species considered for FTD in the invaded and pristine communities, an apparently healthy mature individual, growing in approximately “average” conditions (i.e., not too shaded, infected by pests), was chosen for RNA sampling and FGD analyses. This individual was not included in the FTD measurements due to the destructive nature of sampling leaf tissue required for FGD data. Leaf tissue was collected from focal species across both communities over the course of four days following the field protocol for phylotranscriptomic studies detailed in Yang et al. (2017). Using nitrile gloves changed between each collection, approximately 0.1 g of tissue from mature leaves of a single individual was sampled, immediately placed in a 2‐μl Safe‐Lock Eppendorf Biopur tube (Eppendorf AG), then flash‐frozen in a thermos of liquid nitrogen in the field until samples could be stored at −80°C. For congeneric alien–native species pairs and native species found in both communities, leaf material was collected as close to the same time of day as possible to control for temporal variation. Otherwise, sampling across the invaded and pristine plots was random across time of day and date sampled. After all FTD and FGD sampling, one additional individual representing each focal species was pressed in the field, dried (Blanco et al., 2006), and deposited as a voucher in the Stillinger Herbarium at the University of Idaho (see Table S1 for voucher information and Table S2 for RNA sampling information). These vouchers represent each species sampled in our study, but they are not included in the analyses because FTD and FGD measurements required tissue destruction.

RNA extractions were conducted at the Laboratoire d'Ecologie Alpine (LECA) in France using a protocol developed for plants from diverse taxonomic groups (Yockteng et al., 2013). After removal of DNA with a Turbo DNA‐free Kit (1907M; Ambion Life Technologies), samples were shipped on dry ice to the University of Idaho (USA). A nanodrop was used for an initial general assessment of RNA extraction. For samples that were positively extracted, a Qubit fluorometer was used for more precise quantification of RNA, and a Fragment Analyzer (Advanced Analytical Technologies) was used to assess quality. Using the RNA extraction with the highest quality, RNA‐seq libraries were prepared with a KAPA Stranded mRNA‐Seq Kit (KR0969, v. 3.15) and a 200 (base pair)‐bp insert size. Paired‐end 100‐bp RNA‐seq was performed on Illumina HiSeq 4000 platform at the Vincent J. Coates Genomics Sequencing Laboratory (GSL) at the University of California, Berkeley, with each sample split across two sequencing lanes. RNA‐seq reads were demultiplexed, and low‐quality reads (phred score <20) were removed using the SnowWhite pipeline (Barker et al., 2010; Dlugosch et al., 2013).

From cleaned RNA‐seq reads of each individual sample, transcripts were assembled using SOAPdenovo‐Trans v. 1.04 (Xie et al., 2014) with kmer = 57 (Marx et al., 2021). For the differential expression (DE) analyses (DE of natives growing in the invaded versus. pristine community), the native species collected from each community were pooled to assemble a reference transcriptome (*N* = 2 samples) using the same assembly tools. De novo transcriptomic assembly quality was assessed with basic alignment summary metrics (e.g., number of scaffolds, mean scaffold length, N50), by mapping raw reads to each assembly with bowtie2 v. 2.3.4.2 (Langmead & Salzberg, 2012) to quantify raw read support, and by blasting each transcriptome to universal single‐copy orthologs for embryophyta (embryophyta_odb9) using the program BUSCO v. 3 (Simão et al., 2015) to quantify completeness (creation date: 2016‐02‐13, number of species: 30, and total number of BUSCO groups searched: 1,440).

To determine the putative biological functions of expressed genes, each sample transcriptome was annotated to curated gene ontologies. Scaffolds of unigenes were blasted against annotated *Arabidopsis thaliana* transcripts from the Araport11 blastset (201606) downloaded from TAIR (Swarbreck et al., 2008) following the pipeline outlined in Mandáková et al. (2017). The best hit for each unigene to an *Arabidopsis* gene model was retained. These unigene annotations were then compared with a database of functional annotations for *Arabidopsis* genes (Berardini et al., 2004; Harris et al., 2004) (updated 10 February 2020) to incorporate terms from the Gene Ontology Consortium controlled vocabulary (Harris et al., 2004), including 47 Gene Ontology (GO) slim categories. GO slim categories provide a standardized classification of the biological function of gene products derived from the more detailed gene product attributes defined by the Gene Ontology, and these fall within three broad aspects: biological processes, cellular components, and molecular functions.

Functional genomic diversity was then measured in a few ways. First, we quantified the overall number of distinct unigenes (including gene models) recovered from each sample. Second, we summarized the total number of unigenes matching each functional GO slim category. Third, for the natives found in both communities we quantified differential expression (DE) of genes when growing in the invaded community compared with the pristine community (i.e., occurring with and without the presence of alien species) using the Tuxedo pipeline (Trapnell et al., 2012) with default parameters as follows: Scaffold sequences from the de novo reference transcriptome were indexed with bowtie2‐build (Langmead & Salzberg, 2012). Cleaned read pairs from each sample community (invaded or pristine) for a native species were synchronized using fastq‐pair v. 0.3 (Edwards & Edwards, 2019) and mapped to the native species reference transcriptome with TopHat2 v. 2.1.1 (Kim et al., 2013). Novel transcripts were assembled from reads with Cufflinks v. 2.2.1 (Trapnell et al., 2010). Finally, Cuffdiff v. 2.2.1 (Trapnell et al., 2013) was used to quantify DE by first mapping and then quantifying the abundance of RNA‐seq reads distributed across transcripts (which can include isoforms from alternative splicing). Within‐sample feature lengths and library size effects were normalized by fragments per kilobase of exon model per million mapped reads (FPKM), and log 2 fold differences in normalized read counts between the samples from the invaded and pristine communities were quantified. Only loci with >10 read alignments were considered (‐‐min‐alignment‐count), and read counts were fit to a negative binomial distribution with a blind dispersion method (i.e., each sample was treated as a replicate of the global expression for a species) to specify expected variation at each locus. Significantly differentially expressed transcripts would have greater variance in one of the communities than expected if they were from the same community. A *t* test was used to calculate a *p*‐value, which was adjusted to maintain a false discovery rate of 0.05.

Differential expression (DE) of unigenes between the invaded and the pristine community was summarized by (1) the median absolute value of log 2 fold change in unigene expression with singularly expressed transcripts removed, and (2) the fraction of significantly differentially expressed unigenes (number of significantly differentially expressed unigenes/total unigenes recovered). DE was visualized with the R package cummeRbund (Goff et al., 2013).

### 2.4 Statistical analyses of diversity patterns

We used principal component analysis (PCA) to compare the general distinction between alien and native species provenance and establish overall variation within functional space. Within phenotypic trait functional space (FTD), we compared the variance of measured trait values across species. Within genomic functional space (FGD), we compared the variance of the total number of distinct unigenes matching each functional GO slim category. PCAs of FGD were also conducted separately for the three GO slim aspects, namely biological processes, cellular components, and molecular functions, and combined with tests of multivariate analyses of variance (MANOVA) to assess whether alien and native species showed distinct patterns of FTD and FGD.

To assess differences in functional diversity patterns at the community level, we inferred a classical estimator of functional dispersion for phenotypic and genomic traits separately. This estimator quantifies the average distance between distinct species in a PCoA space based on Euclidean distances computed between all species (Laliberté & Legendre, 2010), using phenotypic trait values and unigene counts for the 47 GO slims as input traits. Functional dispersion (FDis) thus describes the average of functional distances between all species and accounts for abundance by shifting the distance centroid toward more abundant species. It is also important to note that this estimator is by construction unaffected by species richness. The rationale of this estimator is to compute functional diversity in multivariate space defined by nonredundant functional axes (as produced by the PCoA). These estimators of FTD and FGD were computed with the R package FD (Laliberté & Legendre, 2010) separately for the pristine and invaded communities. FGD estimates were also produced for each GO slim aspect separately, again for the whole pristine and the whole invaded community separately.

For comparison of FGD profiles between the five alien–native congeneric pairs in the invaded community, we quantified the number of unigenes (i.e., uniquely assembled gene transcripts, including isoforms) that were annotated to the three biologically meaningful GO slim functional categories (biological processes, cellular components, and molecular functions) for each species, and compared the proportion of unigenes expressed in each category between the alien and native congeneric species pairs. Fisher's exact test of contingency tables was used to identify GO slim aspects that had significantly different proportions of unigenes.

For native species shared between the two communities, linear regression was used to test whether changes in measures of DE (log 2 fold change in unigene expression or the fraction of significantly differentially expressed unigenes) correlated with the difference in relative abundance of each native species in the invaded community compared with the pristine community. All statistical analyses were conducted in R version 3.5.1 (R Core Team, 2018).

## 3 RESULTS

A total of 59 species were encountered across both communities. The pristine community had a greater observed species richness (*N* = 39) than the invaded community (*N* = 36; 28 native and 8 alien), and there were 16 native species shared between communities (Figure 1a). However, many of the species in the pristine community were locally rare, so we focused on dominant species for functional diversity analyses. This resulted in 17 species sampled from the pristine community and 21 from the invaded community (28 total species and 10 shared native species; Table S1). The sampled species covered more than 60% species cumulative abundances in both the invaded and the pristine communities.

A total of 330 Gb of RNA‐seq reads were sequenced. On average, 16.73 million raw paired‐end reads were recovered per sample and cleaning resulted in an average depth of 14 million read pairs (Table S2). Sample transcriptomes had on average 70,123 scaffolds and mean length of 417 bp. An average of 93.30% of raw reads aligned to each sample transcriptome assembly. One sample from the invaded community (*Deschampsia caespitosa* I4_160) and one sample from the pristine community (*Pinguicula vulgaris* P2_47) assembled poorly based on scaffold statistics and BUSCO summaries (Figure S1a) and were removed from downstream analyses. This left a total of 26 unique species sampled for functional and genomic diversity comparisons: 20 species (7 alien and 13 native) from the invaded community and 16 species from the pristine community, including five congeneric alien–native species pairs and 10 native species shared between the two study communities.

After annotation, 28,063 distinct unigenes were found in both communities (Figure 1b). Following the greater species richness in the invaded community, we found greater FGD in the invaded community, with 3,103 distinct unigenes compared with 2,053 in the pristine community (Figure 1b; Table S3). There was also a greater diversity of unigenes within each GO slim in the invaded community (Figure 1c). Separating the alien species from the natives in the invaded community, we see that most of the unique functional genomic diversity was contributed by native species (1,600 unigenes) rather than aliens (807 unigenes) in the invaded community (Figure 1b; Table S3).

**FIGURE 2:**
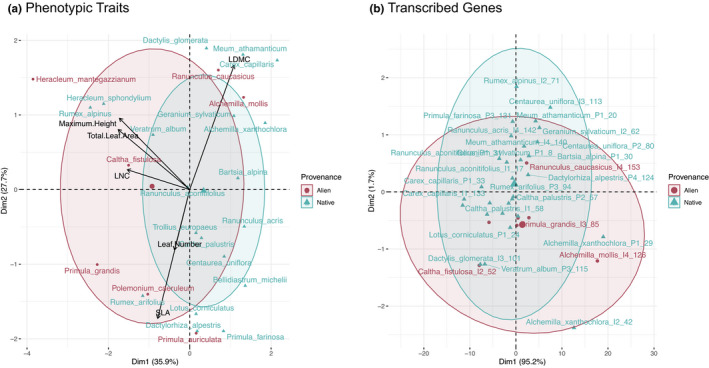
Overall comparison of phenotypic functional trait and functional genomic trait differences for alien (pink) and native (blue) species. Principal component analysis (PCA) showing dispersion of (a) functional phenotypic traits (FTD), and (b) functional genomic traits (FGD), defined by the number of unigenes associated with each GO slim category

Overall, measurements of FTD showed that alien and native species were phenotypically different (MANOVA test *p*‐value = .005). Alien species were generally taller, had more leaves, and had greater total leaf area than native species across the community (Figure S3a; Table S4). The PCA showed phenotypic traits (FTD) discriminate between aliens and natives on the first three planes (axis 1 = 35.82%, axis 2 = 27.75%, and axis 3 = 18.08%; Figure 2a). Compared directly with their native congener, alien species were also generally taller, with more leaves and greater leaf area (Figure S3b). Alien congeners did have lower SLA than their native congeners, except for the introduced *Heracleum mantegazzianum*, which had a greater SLA than its native pair (*Heracleum sphondylium*). Otherwise, LDMC and LNC did not differ between provenance or congeneric alien–native species.

A PCA of FGD data (number of total unigenes matching each functional GO slim category) for each species did not show any batch effect resulting from variation introduced from sampling date or time separating either community type (invaded or pristine) or species provenance (Figure S2). However, there was also no apparent difference in FGD between alien and native species (axis 1 = 95.2%; Figure 2b). Within the invaded community, FGD also does not discriminate between aliens and natives (axis 1 = 94.8%; Figure S4b) nor between alien and native species in congeneric pairs (Figure S5a,b). Within separate GO aspects, MANOVA tests suggest that there are a slight but not significant cryptic difference between FGD of aliens and natives for transcripts annotating to the “cellular components” aspect (*p*‐value = .2367; Figure S6b), and a significant difference for transcripts annotating to the “molecular function” aspect (*p*‐value = .003; Figure S6c; Table S5).

**FIGURE 3:**
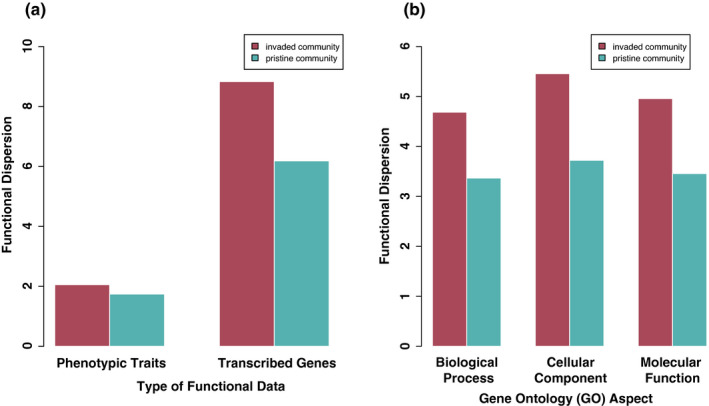
Patterns of functional dispersion (*y*‐axis; unitless) calculated according to Laliberté and Legendre (2010) within the invaded (pink) and pristine (blue) community from (a) the perspective of different types of functional data, transcribed genes (FGD), and phenotypic traits (FTD), and (b) of transcribed genes within each GO aspect

**FIGURE 4:**
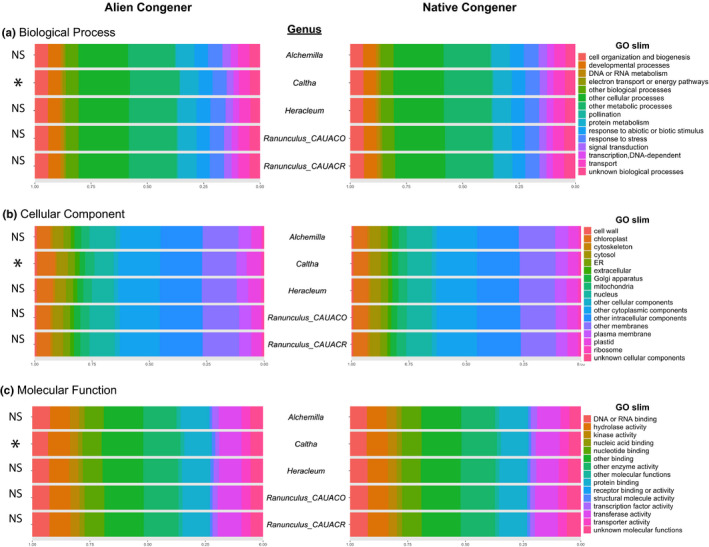
FGD profiles for alien–native congeneric species pairs showing the proportion of distinct unigenes identified within each GO slim category (a‐c): *Alchemilla mollis* (alien)*–Alchemilla xanthochlora* (native)*; Caltha fistulosa* (alien)*–Caltha palustris* (native)*; Heracleum mantegazzianum* (alien)*–Heracleum sphondylium* (native)*; Ranunculus caucasicus* (alien)*–Ranunculus aconitifolius* (native)*;* and *Ranunculus caucasicus* (alien)*–Ranunculus acris* (native). Results from Fisher's exact test for significant differences between the proportion of unigenes in native versus alien congeners are reported to the left of each panel (NS = not significant; *p‐value < .05)

We found consistently greater functional dispersion from the perspective of both FTD and FGD in the invaded community relative to the pristine community (Figure 3a). This result was consistent across all three GO aspects for FGD (Figure 3b). GO slim categories additionally showed how FGD compared between species in alien–native congeneric pairs (Figure 4). There were significant differences in the proportion of distinct unigenes within GO aspects between the alien and native *Caltha* congeners (Figure 4). In general, the alien species showed greater diversity of unigenes for the genera *Alchemilla* and *Ranunculus*, and the native species showed greater diversity for the genera *Caltha* and *Heracleum* (Table S6).

**FIGURE 5:**
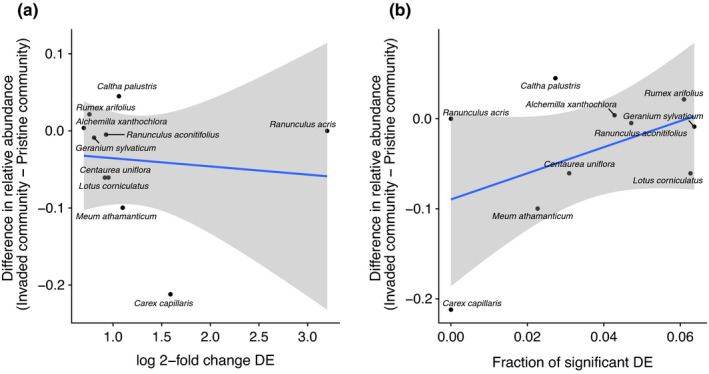
Relationship between differential expression (DE) measured by (a) the absolute value of median log 2 fold change in unigene expression (*y* = −0.01062*x* − 0.02486, adj. *R* ^2^ = −0.1125, *p*‐value = .7717) or (b) the fraction of significantly differentially expressed unigenes (*y* = 1.45037*x* − 0.08958, adj. *R* ^2^ = 0.1152, *p*‐value = .1787), and the difference in relative abundance of each native species grown in the invaded versus pristine communities

Reference transcriptomes assembled for DE of native species sampled in both communities had an average of 129,955 scaffolds, mean scaffold length of 382 bp, and 95.46% alignment rate for raw reads (Table S7). More than 50% of BUSCOs were complete or partial for all species except for *Carex capillaris* (Figure S1b). Although not significant, different trends emerged for the two different measures of DE for native species occurring in both communities (Figure 5). The magnitude in shift of log 2 fold change in DE tended to be lower for natives with a greater relative abundance in the invaded community compared with the pristine community (Figure 5a; *y* = −0.0106*x* − 0.0249, adj. *R* ^2^ = −0.1125, *p*‐value = .7717). However, the fraction of significant DE gene transcripts was positively correlated with the difference in relative abundance between communities (Figure 5b; *y* = 1.4504*x* − 0.0896, adj. *R* ^2^ = 0.1152, *p*‐value = .1787). Removal of the outliers (*Ranunculus acris* and *Carex capillaris* for log 2 fold change and *C. capillaris* for the fraction of significant DE) did not alter results qualitatively (Figure S7; log 2 fold change: *y* = −0.1411*x* + 0.1068, adj. *R* ^2^ = 0.0285, *p*‐value = .3144; fraction of significant DE: *y* = 0.1394*x* − 0.0238, adj. *R* ^2^ = −0.1380, *p*‐value = .8672).

## 4 DISCUSSION

Biological invasions are one of the most visible signatures of human‐mediated impact (Essl et al., 2011; Seebens et al., 2017; Simberloff et al., 2013), and in the face of accelerating global change, it is of both ecological and economic importance to predict which species are most likely to become invasive and their potential for future spread (Bellard et al., 2013). In this study, we aimed to explore transcriptomic diversity for understanding invasion dynamics in an alpine streambed ecosystem. Transcriptomes assembled from RNA‐seq data have the capacity to provide a detailed perspective on functional genomic diversity (FGD) by investigating patterns of the number of distinct transcribed genes (unigenes) and their annotations to biologically meaningful functional (GO slim) categories, or the magnitude of differential gene expression. When used in the comparative framework that has been established for phenotypic functional trait diversity (FTD) in invasion biology, we anticipated this novel axis of biodiversity could illuminate underlying traits that are cryptic or challenging to detect and measure at the phenotypic level. Such an approach is still in its infancy, and the purpose of the present work was to present a proof of concept through a pilot study. Below, we discuss how our findings provide interesting insights into the naturalization and spread of alien species into an alpine riparian community, considering the current limitations of community transcriptomics and how these can be advanced with future research.

It is necessary to keep in mind that while the sampled species richness of the pristine community was lower than the invaded community, the initial survey of the pristine plot showed a greater species richness. However, this richness was driven by many locally rare species at low abundance that we could not destructively sample for this study. Therefore, conclusions of results used to assess hypotheses of invasion dynamics in this system follow from the dominant (in terms of abundance) members of both communities.

### 4.1 Alien species contribute functional diversity at the community level

A primary result of our study is that the naturalization of introduced alien species has not caused a decrease in functional diversity in the invaded community compared with the pristine, as would be expected under a scenario of super dominance of highly competitive species (Mayfield & Levine, 2010). We found greater functional dispersion in the invaded plot (in terms of both FTD and FGD) when accounting for species abundances (Figure 3a), which held for all different gene categories within plant transcriptomes (Figure 3b). Mirroring the functional dispersion results, we found more expressed unigenes in the invaded than the pristine community (Figure 1b), independently of differences of species richness between the communities. Comparing FGD in the invaded alpine community between provenance categories showed that aliens are adding new traits or trait combinations: specifically, 807 additional unigenes that were not expressed in native species from either community (Figure 1b). Removing the addition of FGD from the alien species, the invaded community still had slightly more distinct unigenes than the pristine community (*N* = 243; Figure 1b), reflecting functional differences across the native community itself. Overall, alien species increased the species richness along with the functional diversity of the community, suggesting these species introduced novel traits with different functions.

As for what this novel diversity looks like, mean values of phenotypic functional traits showed differences between provenance categories (Figure S3) and PCA eigenvectors indicated FTD dispersion directed toward maximum height, total leaf area, and LNC traits (Figure 2a). FTD results suggest that novel phenotypes such as greater plant stature and larger total leaf area have favored the local invasion of alien species, in comparison with local native species; this pattern was particularly evident when focusing on congeneric pairs of alien–native species (Figure S3). Patterns of gene expression confirm functional space expansion in the invaded community. Genomic functional traits did not distinguish between provenance as clearly but there was still clustering between aliens and natives across the 47 FGD GO slim features (Figure 2b), which was mainly driven by greater expression of genes related to molecular function (and less significantly cellular components; Figure S6b). As far as we know, this is the first study to compare transcriptomic diversity across nonmodel native and alien plant species in a natural ecological community. However, our FTD results are in line with other studies in comparable community types. While at a global‐scale communities postinvasion have shown mixed trends in patterns of species richness (Peng et al., 2019) and functional trait diversity (Pyšek et al., 2012), previous studies of European flora have found increased observed species richness following invasion (Winter et al., 2009) and functional trait dispersion of alien species compared with native species (Divíšek et al., 2018; Hejda & de Bello, 2013) as we do here. In this system, we find invaders from different phylogenetic lineages bringing novel phenotypes and functions, and gene expression patterns corroborate this with a greater diversity of genes. Therefore, patterns of both FTD and FGD converge in suggesting that niche differences, rather than only competitive fitness differences, are likely an important driver of plant naturalization in our study of an alpine streambed ecosystem.

### 4.2 Congeneric species exhibit functional genomic similarity, but differences in certain phenotypic traits

Congeneric species pair comparisons of diversity within the invaded community have the potential to pinpoint critical functions driving invasion success. Overall, congeneric FTD mirrored differences in FTD between provenances—alien congeners were taller, had more leaves, and had a greater leaf area, while SLA, LDM, and LN were essentially equivalent (Figure S3b). Despite differences at the phenotypic level, largely similar FGD profiles found between alien and native congeners (Figure S8) show that species generally express the same set of genes. This would suggest that phenotypic convergence resulting from habitat filtering may be a primary mechanism for alien establishment success in this community. Some alien and native congeneric species showed altered proportions of unigenes expressed within different biologically relevant aspects, but this was idiosyncratic and significant shifts within GO aspects were only found between *Caltha fistulosa* (alien) and *Caltha palustris* (native; Figure 4). Other studies of transcriptomic profiles between congeneric plant species have found differences between alien and native counterparts. For instance, significant up‐regulation of gene associated with photosynthesis, energy metabolism, protein modification, and stress response was found in the invasive *Mikania micrantha* (Asteraceae) compared with two native congeners (Guo et al., 2018), suggesting expression regulation may facilitate niche occupation or adaptation to novel environments in this invasive species.

It is important to acknowledge that for this type of transcriptomic comparison between closely related alien and native species, our study only offers a very limited statistical power (with only five congeneric pairs) and should just be considered as a proof of concept. We think more studies of this kind would be useful. Measuring and annotating transcriptomic diversity in congeneric native and alien species, with varying degrees of ecological dominance, and in a number of different ecological and evolutionary contexts, could provide novel insights into the functional differences between alien and native species, and into the adaptive genes driving invasive potential in introduced species.

### 4.3 Differential expression of native species collected from each plot

We found two interesting trends when comparing gene expression patterns for native species occurring both in the invaded and in the pristine community with their difference in relative abundance. The magnitude of change in gene expression across the genome was not widely altered (Figure 5a). This could be expected for plant individuals experiencing novel and seemingly more competitive alien neighbors, as suggested by their taller stature and larger leaf canopy. However, while not significant, a positive trend was found between the difference in relative abundance and the fraction of significantly differentially expressed unigenes (Figure 5b). Native species that were less abundant in the invaded communities showed a low fraction of significant DE. As the fraction of significant DE between communities increased, abundances became comparatively greater in the invaded community, suggesting species that respond to invasion through altered gene expression are able to coexist with invaders, and even become more dominant, in the presence of alien species. This lends support to the hypothesis that trait‐based processes (here seen through the lens of functional genomics) impact native community structure. But it is important to keep in mind that in this natural experiment, the species composition of the native community is not completely equivalent between the invaded and pristine communities (i.e., certain native species are not represented in the invaded community), so we cannot definitely conclude that these patterns are driven only by the presence of the invaders. There is the possibility that other native species unique to each community, or unmeasured environmental differences, could also be impacting differential expression patterns observed in co‐occurring natives. Still, there is a good chance that these changes in gene expression patterns in native species are primarily driven by the local presence of alien species, as these are dominant in the invaded study community. In any case, native species did express different genes at different levels in the invaded community, and there was a positive (though not significant) trend with their relative abundance. This suggests an interesting mechanism: That the *plasticity* of gene expression in native plant species encourages persistence in the face of locally invading alien plants. Future work in a study designed within a controlled common garden could be used to corroborate our results and further assess the relationship between FGD and community composition. It is tempting to propose that, as sequencing ease and capacity increases, the description of an organism's transcriptome could be routinely integrated into experimental studies of plant competition to determine the genetic basis of plastic phenotypic changes of plant species experiencing novel competitors.

### 4.4 Extending the axes of functional diversity

What was accomplished with the addition of FGD? Differences between provenances were established from both types of functional data, but genomic functional traits did not distinguish between provenance as clearly as phenotypic functional traits, at least not at the coarse level of comparison used in this study. While FTD might be biased toward certain traits (Lu‐Irving et al., 2018), there is a large body of literature supporting differences in key phenotypic traits, which have been widely used to capture and compare species' strategies along ecological forces structuring vegetation, namely stress, competition, and disturbances (Lavorel & Garnier, 2002; Westoby, 1998; Westoby & Wright, 2006). It is much more challenging to discern biological meaning from the FGD dataset. Even after distilling transcribed genes into 47 GO slim features, we were only able to vaguely say that differences in FGD between provenance categories seem to be largely driven by the expression of genes involved in molecular function.

Importantly, the results of FTD and FGD at the community level mirror each other, illustrating that FGD was useful for detecting the same patterns of diversity, while providing much richer information at certain levels. We found a greater overall magnitude of functional dispersion at the community level from the perspective of FGD than from FTD (Figure 2a) simply due to greater data dimensionality. The phenotypic traits used in this study focused mainly on vegetative characters, and reduced the dimensions of diversity to a handful of traits assumed to be relevant for survival or success in a particular ecosystem (in this case riparian alpine). With transcriptomes, we are potentially targeting many more functions (or functional genomic “traits”), and importantly, we are agnostic about functional relevance a priori. Nevertheless, it must be acknowledged that the perspective of community transcriptomics resides in how gene annotations will be improved in the future, thus potentially highlighting genes with most ecological importance. With the current state of gene annotation libraries, it remains hard to pinpoint which gene expression levels most influence the outcome of biotic interactions.

Interestingly, our study may show some important directions toward resolving this problem. Transcriptomic differences may be related to the phenotypic differences we observed (e.g., alien species have larger leaves and are taller), but observational biases at the phenotypic level were overcome through FGD. In this study, we limited our description of FGD to overall unigene counts and fractions of unigenes that annotated to GO slim aspects to provide a very general illustration of the diversity of transcribed gene products. Besides unigene counts and differential expression, future studies could measure and summarize other physical details of FGD, such as gene regulatory pathways, gene network modifications, or gene duplications. To interpret these novel expressed functions further, more precise taxon‐specific annotations and knowledge of the genetic pathways associated with physiological response would be helpful, but would require detailed experimentally controlled evo–devo and genome‐wide association studies (GWAS), ideally across different tissue types and multiple developmental stages. Preliminary data gathered from natural surveys such as this set the stage to sort out these details of cryptic FGD in future work in order to provide a deeper understanding of invasion dynamics.

As the study of biodiversity enters a new era of big data (Cornwell et al., 2019), this comparative functional genomic approach holds promise for invasion biology, and community ecology more broadly (Swenson & Jones, 2017). Here, we illustrated how the addition of a novel axis of diversity at the level of functional genomics illuminated cryptic changes in functional composition within a natural community. The use of transcriptomics to understand diversity dynamics in ecology and evolution is in its infancy—especially within a community context—and we anticipate functional genomics will provide important links between molecules and morphology in community ecology. Some extensions of the approach outlined here could include investigating the direction of DE patterns to identify significantly up‐ or down‐regulated genes between closely related species different in their competitive ability, or even between different populations of a single species differing in their exposure to biotic or abiotic stressors. The nested and networked nature of gene families, pathways, and the phenotypes they produce allow for an exploration of the effect of (functional genomic) trait scale on ecosystem functioning (Carmona et al., 2016). If these preliminary findings hold, the distribution of functional genomic profiles and how they change across landscapes could significantly advance our understanding of the genetic basis of dynamics within ecosystems.

We recognize there are notable challenges for quantifying and interpreting differential gene expression with RNA‐seq data (Conesa et al., 2016; Finotello & Di Camillo, 2015) from both biological and technical perspectives (Fang & Cui, 2011; Han et al., 2015; Ward et al., 2012). For instance, the ability to accurately detect biological variation in DE is highly dependent on true biological replicates (Liu et al., 2014; Todd et al., 2016), in which due to the limits of study design, we were not able to implement here. In this study, gene expression was quantified from leaf tissue, and patterns of FGD could differ depending on the organ or developmental stage sampled. As more cost‐effective techniques for quantifying gene expression continue to become available, comparing expression profiles across multiple individuals of nonmodel organisms is becoming more feasible (Marx et al., 2020). Interpretation of FGD patterns rests upon functional annotation of expressed unigenes, yet annotations are based on model organisms, so many taxon‐specific, ecologically relevant genes may not be determined (Todd et al., 2016). Additionally, there are many challenges for accurately annotating genes, such as distinguishing orthologous genes and gene families from paralogs. Improvements upon annotation tools, including phylogenetic approaches to identify one‐to‐one orthologs among transcripts (Huerta‐Cepas et al., 2016; Yang & Smith, 2014), are promising for making future comparisons of homologous functional changes in nonmodel species that could be comparable across diverse taxonomic scales, within and between ecosystems. Many different strategies have been developed for analyses of transcriptomes from RNA‐seq data for nonmodel plants (e.g., da Fonseca et al., 2016; Unamba et al., 2015; Ward et al., 2012), and as more transcriptomes (e.g., One Thousand Plant Transcriptomes Initiative, 2019) and genomes (e.g., Kersey, 2019) are added to the toolbox along with their annotations, these approaches will only improve. We foresee that these recent and upcoming advances of functional genomics will open up new research avenues to explore the genetic basis of ecological dynamics, such as biological invasions, response to climate change, adaptation to pollutants, or any other relevant force currently driving human impacts on the biosphere.

## CONFLICT OF INTEREST

The authors have no conflicts of interest to claim in the publication of this research.

## AUTHOR CONTRIBUTIONS

**Hannah E. Marx:** Conceptualization (lead); data curation (lead); formal analysis (lead); funding acquisition (equal); investigation (lead); methodology (lead); project administration (lead); visualization (lead); writing‐original draft (lead); writing‐review & editing (lead). **Marta Carboni:** Project administration (supporting); writing‐review & editing (supporting). **Rolland Douzet:** Conceptualization (supporting); methodology (supporting); writing‐review & editing (supporting). **Christophe Perrier:** Conceptualization (supporting); methodology (supporting). **Franck Delbart:** Conceptualization (supporting); methodology (supporting). **Wilfried Thuiller:** Funding acquisition (equal); project administration (supporting); resources (supporting). **Sébastien Lavergne:** Conceptualization (supporting); formal analysis (supporting); funding acquisition (equal); methodology (supporting); resources (lead); supervision (supporting); visualization (supporting); writing‐original draft (supporting); writing‐review & editing (supporting). **David C. Tank:** Conceptualization (supporting); funding acquisition (equal); methodology (supporting); project administration (supporting); resources (lead); supervision (supporting); writing‐original draft (supporting); writing‐review & editing (supporting).

## Supporting information

Fig S1‐S8

Table S1‐S7

Figs S1‐S8‐caption

## Data Availability

Raw RNA‐seq reads for all 28 species (38 samples) were deposited on the NCBI Sequence Read Archive (BioProject PRJNA622490: https://www.ncbi.nlm.nih.gov/bioproject/PRJNA622490). Assembled transcriptomes for each species are available on the Dryad Digital Repository (https://doi.org/10.5061/dryad.8cz8w9gqs).
