# Supplementary material for: Can functional genomic diversity provide novel insights into mechanisms of community assembly? A pilot study from an invaded alpine streambed
Source: Ecol Evol. 2021 Aug 4;11(17):12075–91. doi: 10.1002/ece3.7973 (PMC8427620; doi:10.1002/ece3.7973)
Supplement: Supplementary file 1 — Fig S1‐S8 [file ECE3-11-12075-s002.pdf]

Figure S1:

a) BUSCO Assessment Results for Sample Transcriptome Assemblies

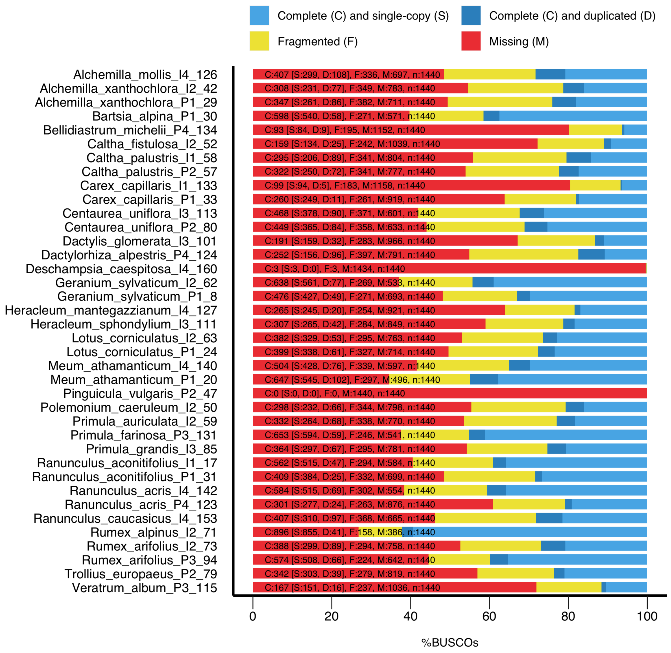

b) BUSCO Assessment Results for Reference Transcriptome Assemblies

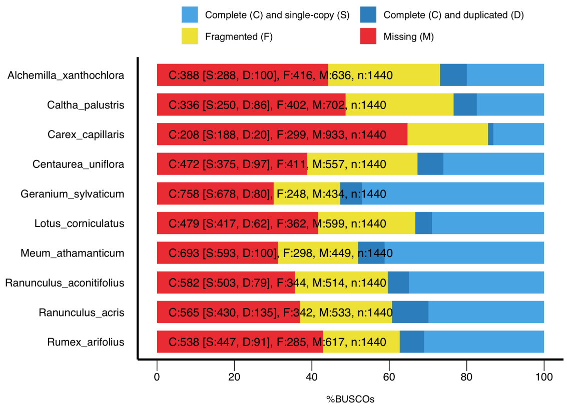

Figure S1: Results from BUSCO assessment of transcriptome assemblies for each sample (a) and the reference assembly used to assess DE for native species sampled from both the invaded and pristine communities (b). Percent of gene content matching universal single-copy orthologs for embryophyta that were complete and single copy (CS), complete and duplicated (CD), fragmented (F), and missing (M) are reported.

Figure S2:

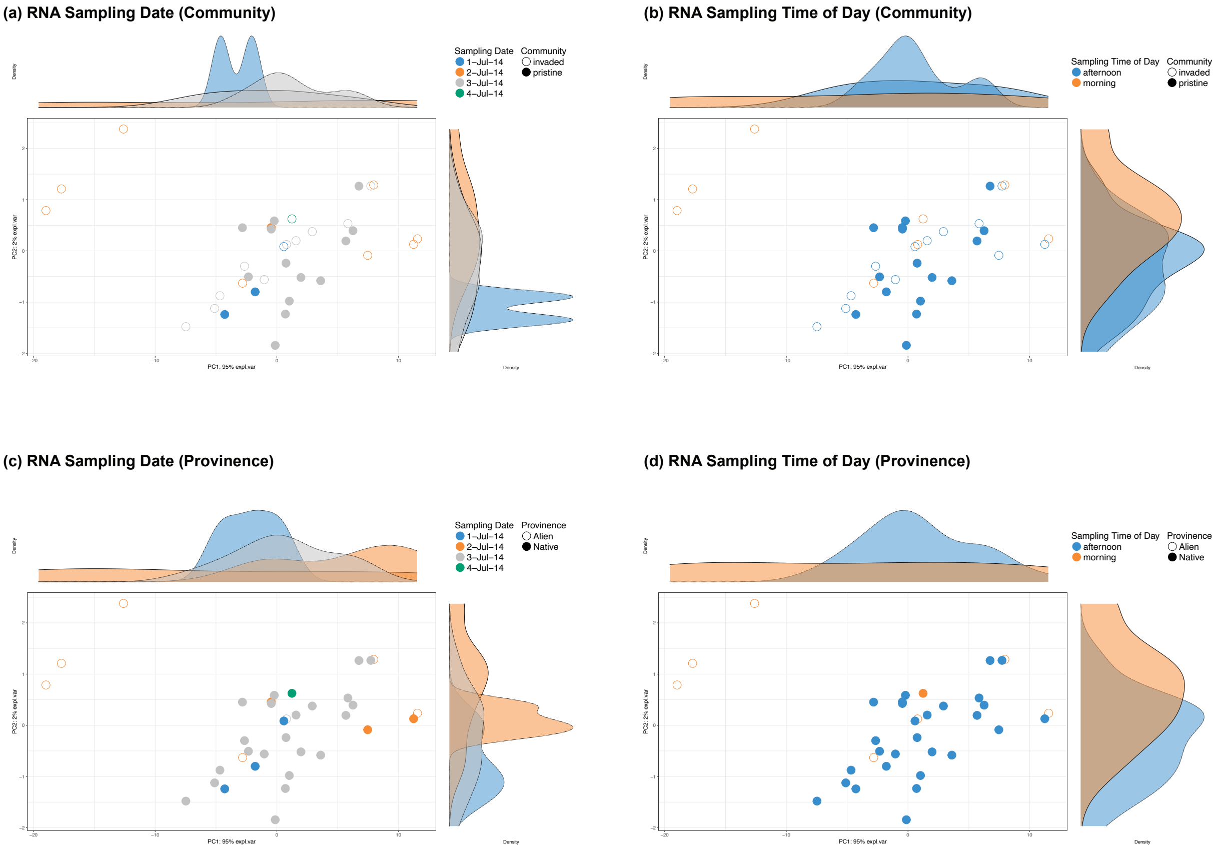

Figure S2: Principal component analysis (PCA) of functional genomic traits indicating RNA sampling date (a, c) and time of day (b, d) distinguished by community type (invaded or pristine) and species provenance (alien or native). Density distributions on each component help to visualize if variation in FGD data resulted from batch effects of RNA collected on the same date or time of day. There is no clear separation in variation on either principal component to indicate potential batch effects.

Figure S3:

(a) Functional Trait Differences: Provenance Category

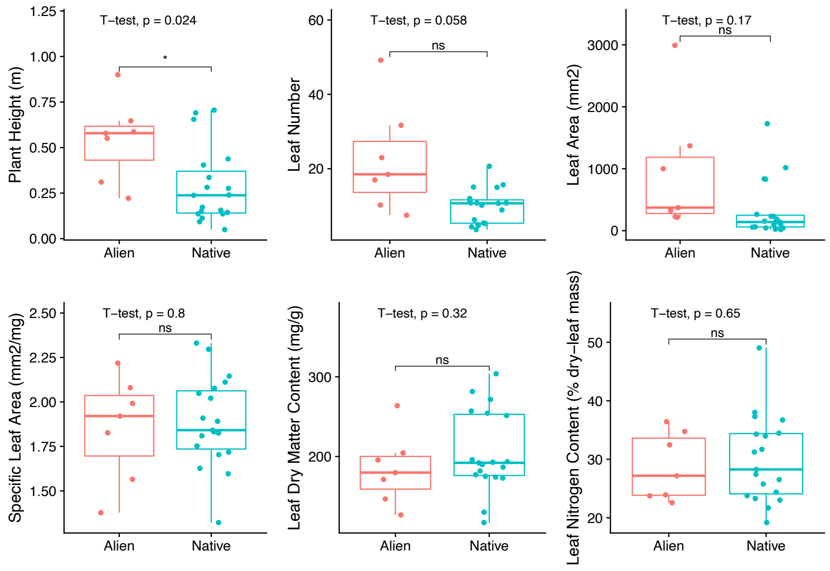

(b) Functional Trait Differences: Alien-Native Congeneric Species Pairs

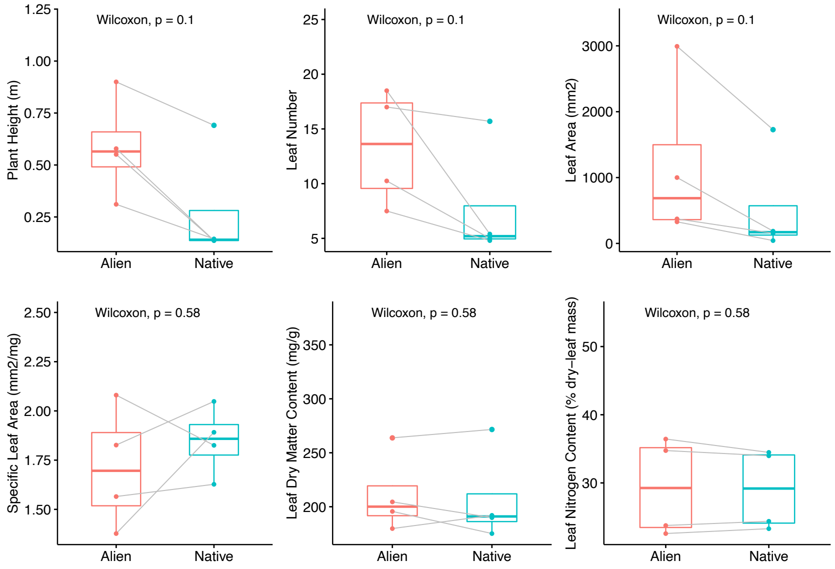

Figure S3: Measurements of trait differences for each functional trait investigated between provenance categories (a) and between alien-native congeneric species pairs (b).

Figure S4:

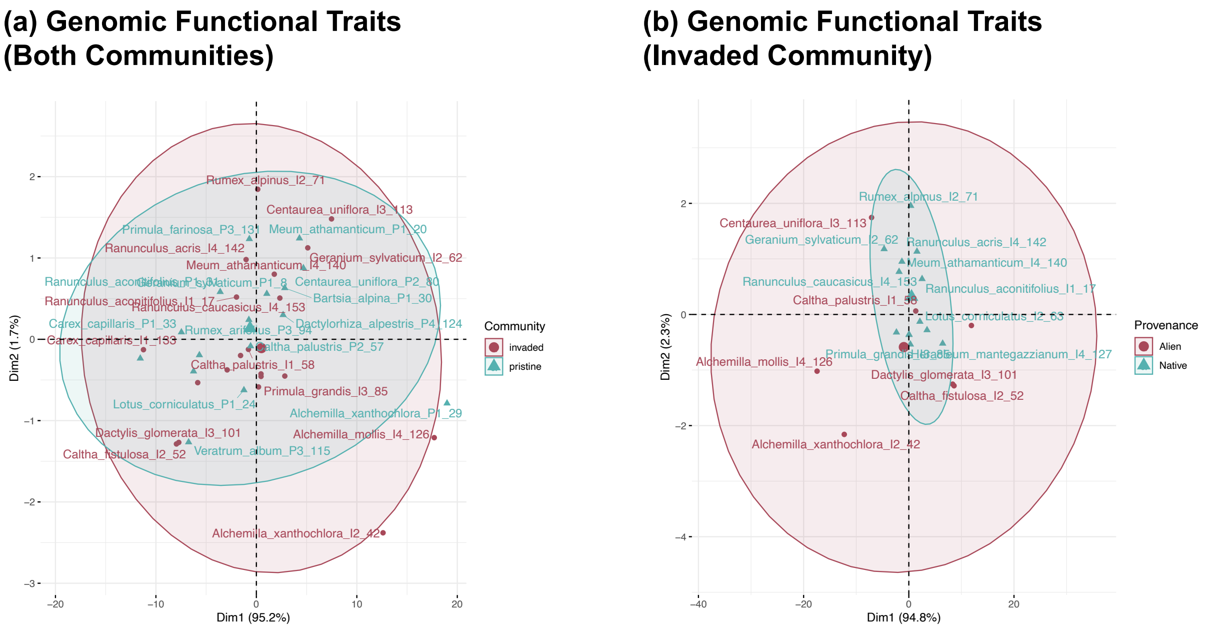

Figure S4: Principal component analysis (PCA) of functional genomic diversity for each sample distinguished by community type (a) and PCA of species collected within the invaded community separately distinguished by species provenance (b).

Figure S5:

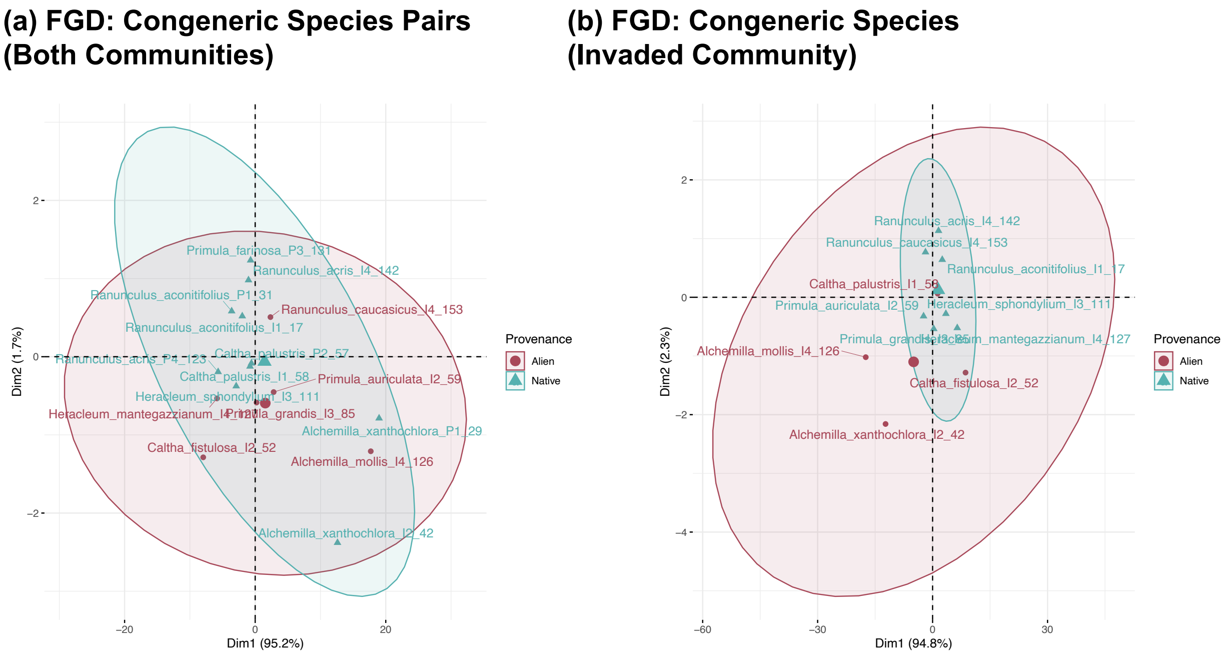

Figure S5: PCA of functional genomic diversity for congeneric pairs of native and alien species overall (a) and within the invaded community separately (b).

Figure S6:

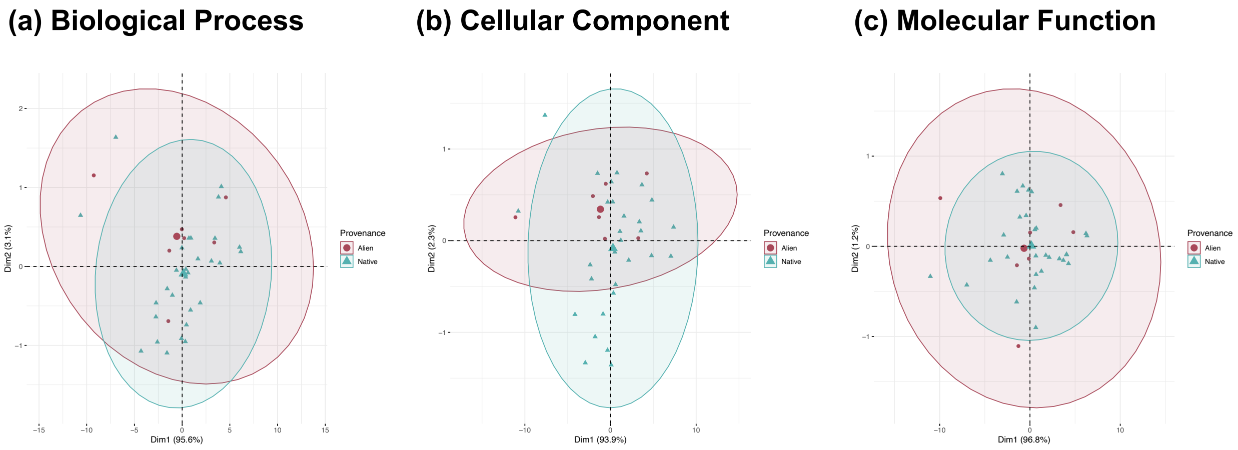

Figure S6: PCA of functional genomic diversity for alien and native species within GO categories: biological processes (a), cellular components (b), and molecular function (c).

Figure S7:

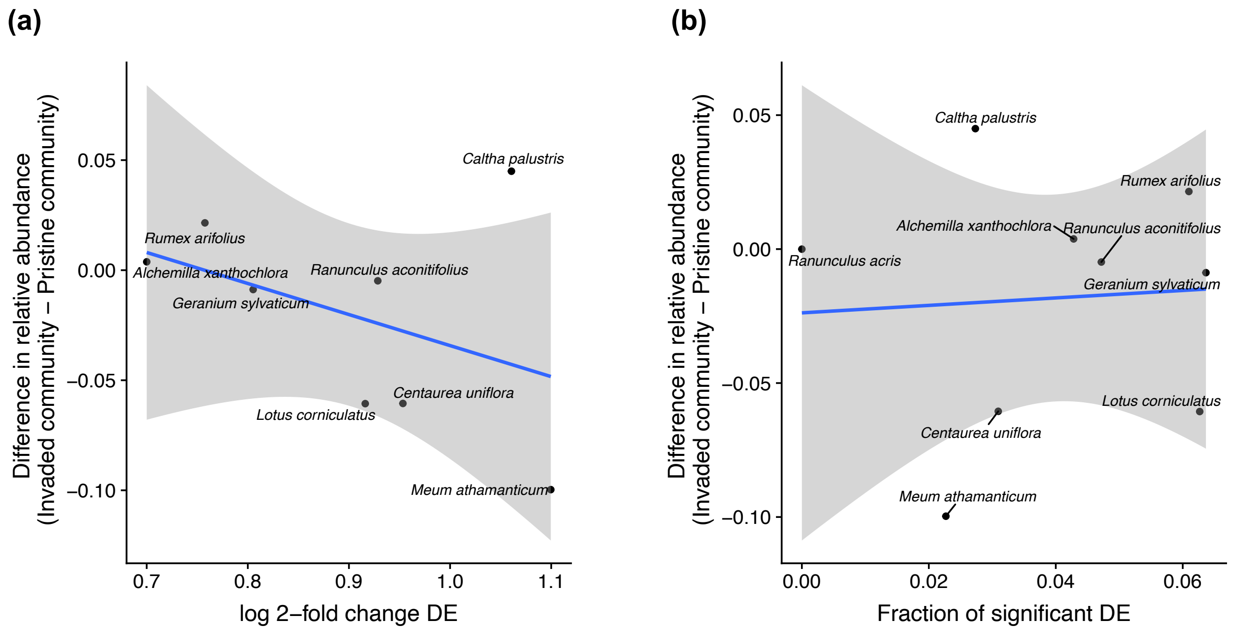

Figure S7: Relationship between differential expression (DE) and the difference in relative abundance of native species grown in the invaded versus pristine communities with outliers removed.

Figure S8:

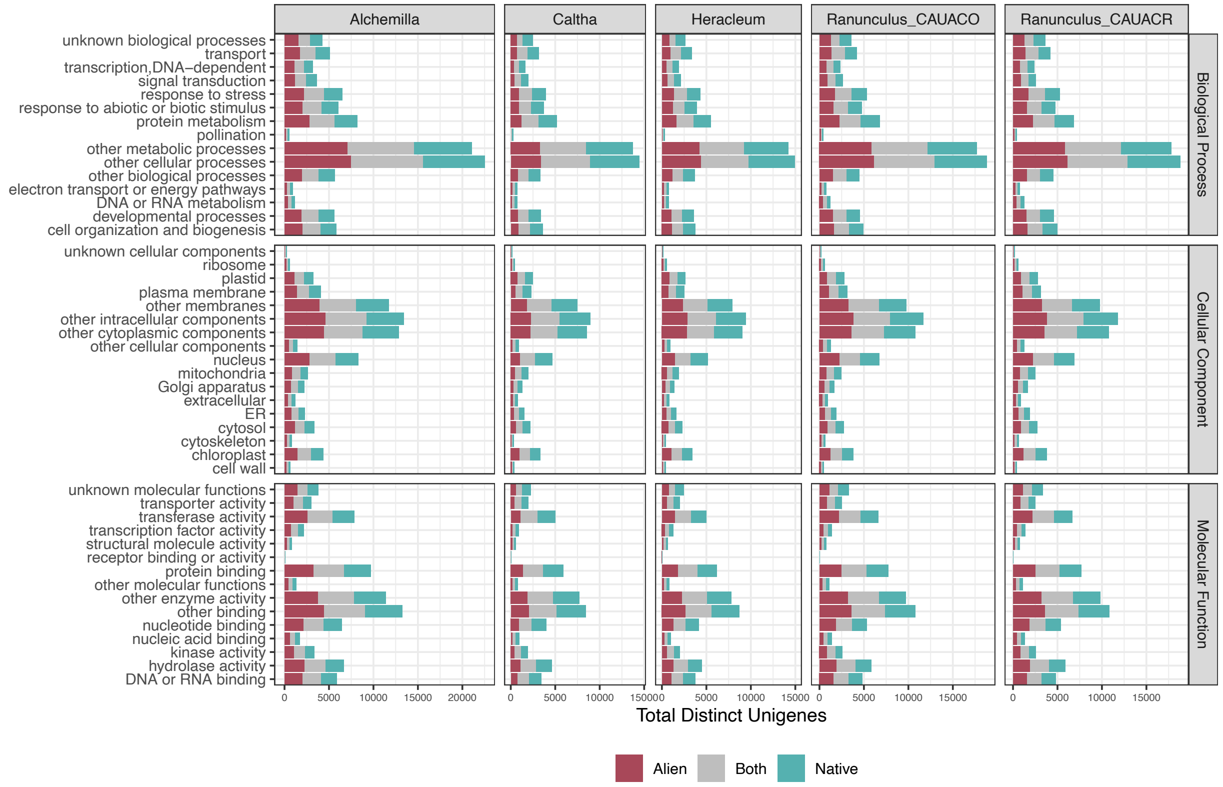

Figure S8: Total number of unique and shared distinct unigenes for each alien (pink) or native (blue) species in a congeneric pair that are associated with GO slim categories: *Alchemilla mollis* (alien) - *Alchemilla xanthochlora* (native)*; Caltha fistulosa* (alien) - *Caltha palustris* (native)*; Heracleum mantegazzianum* (alien) - *Heracleum sphondylium* (native)*; Ranunculus caucasicus* (alien) - *Ranunculus aconitifolius* (native)*; Ranunculus caucasicus* (alien) - *Ranunculus acris* (native).
